# Phytochemicals and Nano-Phytopharmaceuticals Use in Skin, Urogenital and Locomotor Disorders: Are We There?

**DOI:** 10.3390/plants11091265

**Published:** 2022-05-08

**Authors:** Mogana Rajagopal, Alok K. Paul, Ming-Tatt Lee, Anabelle Rose Joykin, Choo-Shiuan Por, Tooba Mahboob, Cristina C. Salibay, Mario S. Torres, Maria Melanie M. Guiang, Mohammed Rahmatullah, Rownak Jahan, Khoshnur Jannat, Polrat Wilairatana, Maria de Lourdes Pereira, Chooi Ling Lim, Veeranoot Nissapatorn

**Affiliations:** 1 Faculty of Pharmaceutical Sciences, UCSI University, Kuala Lumpur 56000, Malaysia; mogana@ucsiuniversity.edu.my (M.R.); leemt@ucsiuniversity.edu.my (M.-T.L.); anabelle@ucsiuniversity.edu.my (A.R.J.); porcs@ucsiuniversity.edu.my (C.-S.P.); 2 School of Pharmacy and Pharmacology, University of Tasmania, Hobart, TAS 7001, Australia; alok.paul@utas.edu.au; 3 School of Allied Health Sciences and World Union for Herbal Drug Discovery (WUHeDD), Walailak University, Nakhon Si Thammarat 80160, Thailand; tooba666@hotmail.com; 4 Biologica Sciences Department, College of Science and Computer Studies, De La Salle University, Dasmarinas 4114, Philippines; ccsalibay@dlsud.edu.ph (C.C.S.); mstorres@dlsud.edu.ph (M.S.T.); 5 Department of Biology, College of Arts and Sciences, Central Mindanao University, Bukidnon 8710, Philippines; f.mariamelanie.guiang@cmu.edu.ph; 6 Center of Biodiversity Research and Extension in Mindanao (CEBREM), Central Mindanao University, Bukidnon 8710, Philippines; 7 Department of Biotechnology & Genetic Engineering, University of Development Alternative, Lalmatia, Dhaka 1207, Bangladesh; rahmatm@uoda.edu.bd (M.R.); rownakj86@bge.uoda.edu.bd (R.J.); jannat.koli.22@gmail.com (K.J.); 8 Department of Clinical Tropical Medicine, Faculty of Tropical Medicine, Mahidol University, Bangkok 10400, Thailand; 9 CICECO—Aveiro Institute of Materials, Department of Medical Sciences, University of Aveiro, 3810-193 Aveiro, Portugal; mlourdespereira@ua.pt; 10 Division of Applied Biomedical Science and Biotechnology, School of Health Sciences, International Medical University, Kuala Lumpur 57000, Malaysia; chooi_linglim@imu.edu.my

**Keywords:** nanomaterials, locomotor disorder, dermal disorder, urogenital disorder, phytopharmaceuticals

## Abstract

Nanomedicines emerged from nanotechnology and have been introduced to bring advancements in treating multiple diseases. Nano-phytomedicines are synthesized from active phytoconstituents or plant extracts. Advancements in nanotechnology also help in the diagnosis, monitoring, control, and prevention of various diseases. The field of nanomedicine and the improvements of nanoparticles has been of keen interest in multiple industries, including pharmaceutics, diagnostics, electronics, communications, and cosmetics. In herbal medicines, these nanoparticles have several attractive properties that have brought them to the forefront in searching for novel drug delivery systems by enhancing efficacy, bioavailability, and target specificity. The current review investigated various therapeutic applications of different nano-phytopharmaceuticals in locomotor, dermal, reproductive, and urinary tract disorders to enhance bioavailability and efficacy of phytochemicals and herbal extracts in preclinical and in vitro studies. There is a lack of clinical and extensive preclinical studies. The research in this field is expanding but strong evidence on the efficacy of these nano-phytopharmaceuticals for human use is still limited. The long-term efficacy and safety of nano-phytopharmaceuticals must be ensured with priority before these materials emerge as common human therapeutics. Overall, this review provides up-to-date information on related contemporary research on nano-phytopharmaceuticals and nano-extracts in the fields of dermatological, urogenital, and locomotor disorders.

## 1. Introduction

Physicians and patients have recognized the use of herbal medicine since ancient times [1]. For instance, the first-ever plant-derived painkiller, morphine which belongs to the benzylisoquinoline class of alkaloid, was isolated from *Papaver* *somniferum* L. (Papaveraceae) and authorized to be used in 1827 [2]. Herbal medicines are well known for their better therapeutic performance as well as lesser side effects compared to modern medicines. The demand for phytochemicals and plant products has been increasing rapidly in many areas of medicine, as in the treatment of dermal, urogenital, and locomotor disorders. Advanced phytopharmaceutical research especially with novel drug delivery systems by applying nanotechnology plays an important role in troubleshooting scientific needs with the determination of the pharmacokinetics, mechanism of action, site of action, accurate dosage, improved bioavailability, and reduced toxicity of various herbal medicines [3,4]. Several safety concerns related to biocompatibility, possible toxicity (of unknown natural compounds), and lack of enough clinical trials on medicinal plants and herbal medicines can be resolved by the implementation of nano-based drug delivery systems [5,6,7]. Thus, herbal medicines can be used for the treatment of a wide range of ailments, including dermal, urogenital, and locomotor disorders.

Nanoparticles are often classified as particles of less than 100 nm in diameter. They occur extensively in nature as products of photochemical, plant, and algae activity and have also been created as by-products of combustion and food cooking for thousands of years [8]. There are various kinds of nanosystems available, such as niosomes, liposomes, nanostructured lipid carriers (NLCs), and nanoemulsions. Niosomes are defined as microscopic vesicles composed of non-ionic surfactants, liposomes as microscopic spherical vesicles having one or more phospholipid bilayer membrane, NLCs as novel nano-sized pharmaceutical formulations composed of solid and liquid lipids, surfactants, and co-surfactants. Nanoemulsions as nano-sized emulsions have droplet sizes between 20 and 500 nm, respectively. Nanomedicine is the application of nanoscale materials such as nanoparticles for the diagnosis, monitoring, control, prevention, and treatment of disease [9]. The field of nanomedicine and the application of nanoparticles has been of keen interest in several industries, such as electronics, communications, cosmetics, biology, and medicine [10]. In medicine, these nanoparticles have various attractive properties that have brought them to the forefront in the search for novel drug delivery systems with most advances in the utilization of nanoparticle drug delivery for the treatment of cancer with several nanotherapies being used clinically after approval by the FDA in the United States of America [11,12]. The properties exhibited by nanoparticles include a high surface-to-volume ratio, high surface energy, unique mechanical, thermal, electrical, magnetic, and optical behaviors [13].

The term “nanotechnology” is derived from a Greek word that means dwarf, which employs the concepts of engineering and manufacturing at the molecular level [14]. The advantages generated by the use of nanotechnology can assure the revolutionary changes in herbal medicines along with several other multidisciplinary emerging applications in chemistry and physics. The reason behind the achievements of nanotechnology in medicine includes the possibility of working at the same scale of many biological processes, cellular mechanisms, and organic molecules. For this reason, medicine has looked at nanotechnology for the ideal solution in the treatments of several diseases. Furthermore, the methodology has drawn attention toward providing treatments in a safe and effective form [15].

**Figure 1:**
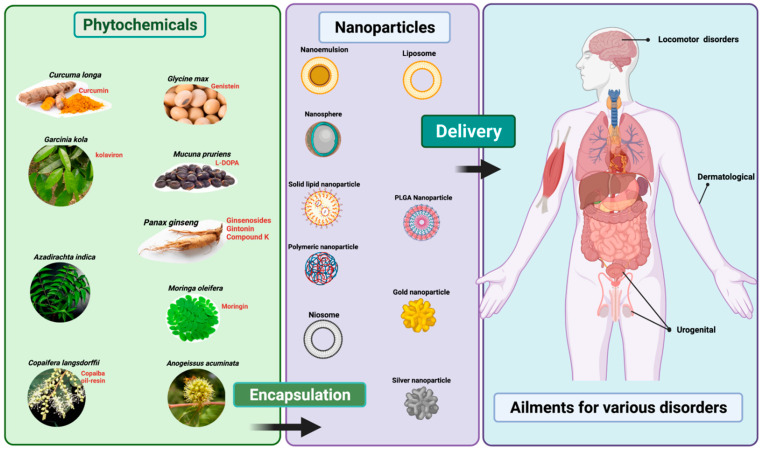
Representation of delivery of phytopharmaceutical using nanotechnology. The figure was made with www.biorender.com (access date: 15 March 2022).

From the existing literature, the increasing trend in nanoformulation using phytochemicals studies has been remarkable, particularly from the last 5 years (from 2018 to date) and it is commonly investigated against cancer-related disorders. Thus, it is timely for us to write a focused review on the current situation of the application of nanoformulations with phytochemicals and herbal medicines. This review therefore focuses on the potential of herbal medicines highlighting the successful application of nanotechnology to treat some diseases, specifically dermal, urogenital, and locomotor activities (Figure 1). In addition, this review aims to understand the justification and significance of using nanotechnology-derived phytochemicals or herbal formulations (i.e., nano-phytopharmaceuticals) in the three specific disorders based on locomotion, skin, and urogenital conditions (Figure 1).

## 2. Materials and Methods

Databases such as PubMed, Medline, Scielo, Thomson Reuters ISI Web of Knowledge, and Science Direct were searched, combining the following keywords: “Nanotechnology”, “plant-based medicine”, “herbal nanoformulations”, “phytochemical-based nanoformulations”, and “nano-phytopharmaceuticals”. In addition, the available scientific literature within the last decade (2011–2022) was considered in this review. The scholarly search engine “Google Scholar”, a search using the keywords “nanoformulation’” and “phytochemicals” showed a total of 5420 publications (without any time limits and citations). The search results also demonstrated that this research field has been growing steadily from 2018 to date, as we found 4980 articles available since 2018. Similarly, using the keywords “nanoformulation” and “herbs” produced a total of 7180 articles (without any time limit) and especially showed an uprising trend of 5630 articles between 2018 and 2022.

## 3. Therapeutic Applications of Nano-Phytopharmaceuticals

### 3.1. Nano-Phytopharmaceuticals in Dermal Disorders

Dermatological disorders are prevalent worldwide and regarded as one of the major global burdens among various diseases [16]. Severe skin damage from burns or wounds as well as acne (i.e., often causes erythematous papulopustular lesions such as rash consisting of papules and pustules) can also lead to trauma and further psychosocial stresses besides possible pain or other aggravations caused by the disorder itself [17,18]. Dermatological disorders can be atopic dermatitis, alopecia (androgenic alopecia and alopecia areata, both indicating hair loss), hirsutism (growth of excess coarse body hair usually in women in places where hair is not supposed to grow), hyperhidrosis (excessive sweating), hidradenitis suppurativa (chronic and progressive inflammatory skin condition affecting groin, buttocks, and perineal and perianal regions), vitiligo, psoriasis, and melanoma [19].

Most dermatological disorders affect the outermost layer of the skin (horny layer), which is typically water repellent and dense, the latter characteristic acting as an effective barrier against rapid passage of any outward items, which may be chemicals or infectious agents. Topical therapeutic agents usually contain in combination the agent and a base-formulation, which facilitates the absorption of the agent. Drugs for dermatological disorders must cross the horny layer to get to the root of skin infection to produce their therapeutic effects. A low molecular weight of the therapeutic agent (20-300 kDa) enhances penetration of the horny layer or stratum corneum [20], which is further enhanced if the agent is applied as an oleaginous ointment, emulsified ointment, cream, or gel. Nanotechnology can be an important tool for the delivery of therapeutic agents for both topical and transdermal applications through engineered nanoparticles of drugs and enabling them to better reach their target sites. Various types of nanoformulations are available such as solid nanoparticles, liposomes, secosomes, transferosomes, ethosomes, niosomes, nanoemulsions (NE), nanostructured lipid carriers (NLCs), solid lipid nanoparticles (SLNPs), and flexible nanovesicles [21].

Solid nanoparticles, such as zinc oxide and titanium dioxide nanoparticles (NPs) are mainly used in sunscreens to filter out UVA and UVB radiations. Studies on keratinocytes suggest that titanium dioxide nanoparticles are safer than zinc oxide, as zinc oxide NPs can generate reactive oxygen species within cells. Both NPs have been found to produce adverse effects in human keratinocytes in vitro following long-term exposure [22]. Liposomes are usually composed of cholesterol and phospholipids that show higher biocompatibility, improved solubility, and efficacy of lipophilic and amphiphilic drugs and thus facilitate the application of topical drugs [23].

Nanomaterials such as NLCs are prepared from a combination of solid lipid (SL) and liquid lipid (LL) ingredients. The use of LL in the manufacture of NLCs permits a greater drug load. The SLs include compounds such as glyceryl monostearate and glyceryl tripalmitate; the LLs include a more diverse variety of compounds such as oleic acid and squalene. Surfactants used in the preparation of NLCs include lecithin and Tween 80 [24]. Flexible or deformable nanovesicles have greater penetrability through biological barriers but thus far have seen limited use because of their physical and chemical instabilities. However, a recent study reported that flexible nanovesicles at a low density and containing 8% lactose and trehalose at a ratio of 1:4 have a spherical shape, smooth surface morphology in the lyophilized state, a whorl-like structure, high entrapment efficiency, and deformability after reconstitution; thus confirming their stability. Importantly, the secondary structure of insulin was well protected in the insulin-phospholipid complex deformable nanovesicles [25], which further confirmed their functional ability.

From the above section(s), it is apparent that nanovesicles and nanoparticles can play an important role in the delivery of drugs to target organs especially on skin. It is important because many drugs have poor aqueous solubility; thus limiting their bio-absorption. These lipophilic drugs can be encapsulated within nanovesicles as nanoparticles and then administered through suitable routes. Various nanotechnological approaches have been and still are experimented with towards a more efficacious treatment of skin disorders. The therapeutic nanoparticles comprise conventional drugs, crude extract of plants, and phytochemicals. For example, the ethanolic extract of *Ocimum* *sanctum* L. (Lamiaceae) reportedly has anti-aging properties on skin, as demonstrated by its anti-oxidant and anti-inflammatory properties, as well as its inhibitory features against hyaluronic acid and collagen fiber degradation inhibition [26]. The encapsulation of the ethanolic extract was completed in several types of nanodelivery systems, including NLCs, NEs, liposomes, and niosomes. Among the various delivery systems containing *Ocimum* *sanctum* L. (Lamiaceae) extract nanoparticles, NLC and NE were the most stable, with NLC delivering the highest amount of extract to the skin layer [27]. The ethosome gel was reported to deliver quercetin to treat inflammation, and amphotericin B to treat fungal infections [28,29]. Quercetin-loaded phospholipid vesicles containing, in addition, 5% polyethylene glycol demonstrated effectiveness in amelioration of skin inflammation induced by TPA (12-*O-*tetradecanoylphorbol-13-acetate). The nanoethosomal formulation exhibited a 3.5-fold higher skin deposition of amphotericin B, leading to a significant increase in anti-fungal activity against *Candida* *albicans*.

Application of various forms of nanodelivery systems for the treatment of skin disorders have been reviewed by Roberts et al. [21]. These include liposome, ethosome, and deformable liposome-based delivery of ketoconazole to treat dermatological fungal infections from *Candida* *albican*; the use of nanostructured lipid carrier-based gel to deliver clobetasol propionate to treat eczema; the use of solid lipid nanoparticles for delivery of artemisone and doxorubicin for the treatment of melanoma and squamous cell carcinoma, respectively. Silver nanoparticles have been used to treat scalp-based fungal infections caused by *Malassezia* *furfur*; and gold nanoparticles are used for the treatment of psoriasis. The use of tyrospheres (tyrosine-derived nanospheres) as a delivery medium for vitamin D_3_ has also proved to be effective for psoriasis treatment. It appears that there is enhanced absorption of vitamin D_3_ through this nano-treatment method [30]. In fact, as reviewed by Petit et al., the use of biodegradable nanocarriers for delivery of vitamin D_3_ or other therapeutics for psoriasis treatment includes nanospheres, nanocapsules, liposomes, ethosomes, solid lipid nanoparticles, and nanostructured lipid carriers [31].

Curcumin, which is derived from rhizomes of *Curcuma* *longa* L. (Zingiberaceae)*,* containing nanomaterials, including lipid-based nanoparticles such as liposomes, niosomes, solid lipid nanoparticles, and nanostructured lipid carriers are used in various dermatological disorders such as psoriasis, dermatitis, bacterial, viral and fungal infections, burns, acne, vitiligo, arthritis, and skin cancer [32,33,34]. Lipid-based nanoparticles (NLCs and SLNPs) of curcumin have higher biocompatibility with skin layers, can increase their penetration into this organ and thus increase their solubility, stability, and therapeutic efficiencies [33] (Table 1). NLCs and SLNPs can also increase patient compliance by maintaining delayed and regulated release and improving their pharmacological activities [35,36].

**Figure 2:**
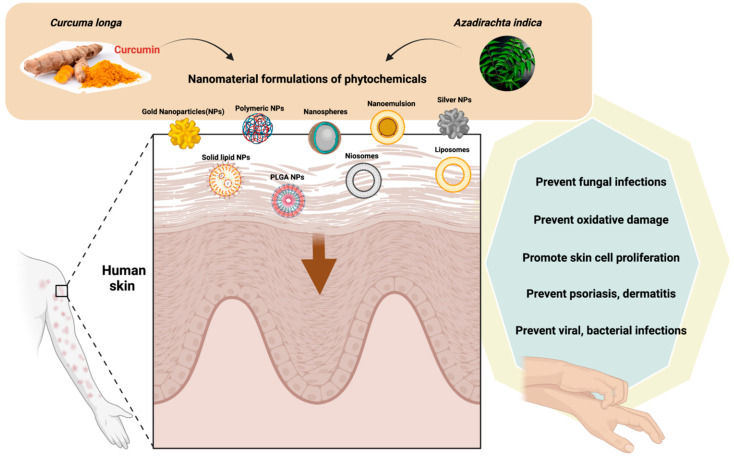
The uses of nanomaterials with phytochemicals of *Curcuma* *longa* and *Azadirachta* *indica* oil in dermatological disorders. The figure was made with www.biorender.com (access date: 15 March 2022).

A phospholipid-based nanoformulation containing neem oil, derived from *Azadirachta* *indica* A. Juss. (Meliaceae), was incorporated in argan-liposomes and argan-hyalurosomes by sonicating with argan oil and soy lecithin in the presence of water, as described by Manca and colleagues [46]. The formulation contained vesicles of 140 nm in diameter with negative charge [46], which protected skin cells from oxidative stress (Figure 2).

Apart from phospholipid-based nanoformulations of neem oil, nanostructures (NSs) have started to show efficacy in healing burns caused by fire or scalding objects. NSs are single or multidimensional nanomaterial-fortified structures within the measurement range of nanometer (10^−9^ m) scale. NSs are classified into two major types, namely organic NSs, which include nanoemulsions, nanogels, liposomes, and so forth, and inorganic NSs containing nanocarbons or silver, copper, or gold nanoparticles (NPs). Nanocarbons can be fullerenes, graphene, or carbon nanotubes. Organic polymeric NPs for burns may contain curcumin, chitosan, and a variety of other substances along with poly lactic-co-glycolic acid (PLGA), the latter acting as a biodegradable and biocompatible copolymer. It has been shown that full-thickness wounds treated with epidermal growth factor (EGF)-loaded PLGA-NPs gave the fastest healing with the highest level of fibroblast production [59]. Considering the increasing use of nanoparticles and nanodelivery systems, it comes as no surprise that 94 patents were published in the area of nanotechnology-based delivery systems as skin penetration enhancers between 2008 and 2018 [60].

Various research studies are on-going on herbal remedies and natural products for effective and safe therapeutics, whereby advancement of novel drug delivery systems with such candidates are in basic and clinical trials. The main requirement is to develop better systems for proper delivery of such drugs at the targeted site. Nanoparticles with use of herbal medicines will significantly increase their potential for treatment of multiple chronic diseases. A number of successful examples with evidence have been presented in the direction of nano-research. It is predicted that beneficial relevance of herbal medicine utilized with nanotechnology will potentially strengthen existing drug delivery systems. Though nano-phytomedicines may promise extraordinary opportunities in the field of drug delivery of conventional and herbal medicines for treatment of various ailments, their safety should not be neglected. The alterations in physicochemical and structural characteristics of synthesized nano-size particles with a reduced size could be responsible for a few material interactions that could lead to toxicological properties. Despite the toxicity, the benefits of applying nanoparticles and nanodelivery systems in cutaneous disorders seem to outweigh the risks and can prove to offer greater benefits in the treatment of disorders such as burns, acne, and a host of other damaging diseases with associated psychosocial problems.

### 3.2. Nano-Phytopharmaceuticals in Urogenital Disorders

**Figure 3:**
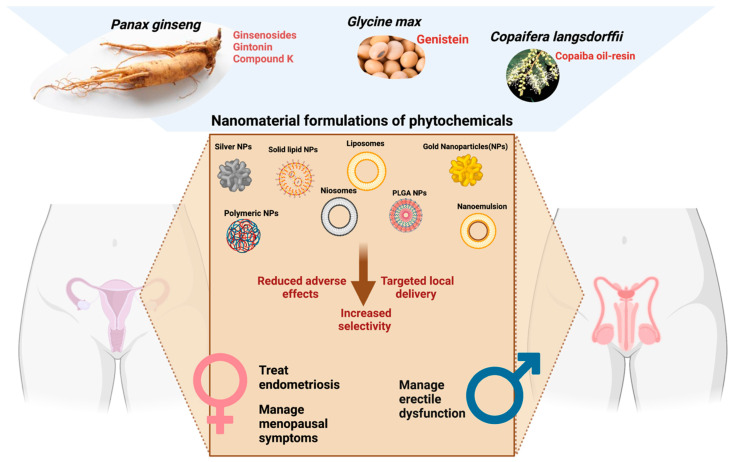
Application of nano-phytopharmaceuticals in urogenital disorders. The figure was made with www.biorender.com (access date: 15 March 2022).

The application of nanotechnology to deliver herbal molecules permits bioactive compounds for targeted site delivery (Figure 3). This application is crucial for the management of menopause as the targeted delivery can minimize the side effects of the herbal product, which contains hormone-like activity. Hormone replacement therapy (HRT) is the primary management strategy for menopause. Although the benefits of using HRT (estrogen and progesterone) for the management of moderate-to-severe menopausal symptoms outweigh the risk, the non-selective delivery of the hormones may cause increased risks of cerebrovascular diseases, such as stroke [61]. Herbal medicines are promising alternatives for the management of menopause. Phytoestrogen is a plant-derived compound that is structurally and/or functionally similar to estrogen. Plant compounds such as soy, red clover, hop, and other botanicals contain naturally occurring phytoestrogens [62]. Genistein is a primary phytoestrogen compound of soybean which is poorly soluble in an aqueous medium. Its poor aqueous solubility and low serum concentration after administration warrant the development of a novel drug delivery system [63]. Encapsulation of genistein in Fe_3_O_4_-carboxymethylated chitosan nanoparticles and EudragitR E cationic copolymers improves water solubility, leading to better absorption from the gastrointestinal tract [63,64]. A low dose of phytoestrogen is associated with the development and progression of breast cancer in vitro and in vivo [65]. Activation of estrogen receptors in the breast by phytoestrogen promotes the growth of breast cancer. These limitations can be overcome with the incorporation of a nanotechnology-based drug delivery system. Encapsulating phytoestrogen in nanoparticles may help delivery of the bioactive compounds to the estrogen receptors in endothelium and vascular smooth muscle specifically. The agonist effect of estrogen receptors on vascular smooth muscle helps to relieve vasomotor symptoms (hot flash, night sweat) in menopausal women. The extended-release activity of the herbal preparation can be achieved through encapsulation into nanocarriers, such as multivesicular liposomes. This approach is valuable in delivering bioactive compounds which are intended to produce long-lasting action. Genistein nanoparticle preparation has been widely used for anticancer therapy [66]. However, its potential as a phytoestrogen to treat menopause is not yet fully elucidated.

Herbal products such as rhizome extract of wild yam (*Dioscorea* *villosa* L. (Dioscoreaceae), root extract of Dong Quai (*Angelica* *sinensis* (Oliv.) Diels (Apiaceae)), evening primrose oil (*Oenothera* *biennis* L. (Onagraceae)), dried root of Maca (*Lepidium* *meyenii* Walp. (Brassicaceae)) are commonly used among menopausal women to relieve menopausal symptoms [67]. Black cohosh (*Cimicifuga* *racemosa* L.) Nutt. (Ranunculaceae) is one of the common herbal products that has been used among indigenous people for the management of menopausal symptoms. Several mechanisms of action of black cohosh have been proposed: selective estrogen receptor modulation, serotoninergic pathway, anti-oxidation, and anti-inflammation [68]. The blood-brain barrier is a challenge for the delivery of bioactive compounds, which act centrally. Formulating black cohosh in nanoparticles may help enhance the crossing of the bioactive compound through the blood-brain barrier. This novel formulation increases the selectivity of black cohosh bioactive compounds towards the central serotoninergic pathway in the brain [69].

Copaiba oil-resin is obtained from *Copaifera* L. species. It is effective against endometrial cell growth. To facilitate the delivery of Copaiba oil-resin towards endometrial derived cells, it has been formulated into nanoparticles using organically modified sodium montmorillonite derivatives as a nanocarrier [70]. Reduction in the proliferation of endometriotic cells in vitro by the nanoparticles of Copaiba oil-resin suggested the promising alternative therapy for the treatment of endometriosis [70] (Figure 3).

When it comes to male reproductive disease, erectile dysfunction is one of the common debilitating conditions which affects aging men. Current management with phosphodiesterase type 5 (PDE5) inhibitor has side effects such as headaches and decreased blood pressure. The side effects become less prominent with increased selectivity of the PDE5 inhibitors [71]. The current areas where nanotechnology can be applied for the management of erectile dysfunction are: (1) topical delivery of drugs for on-demand erection, (2) injectable gel into the penis, (3) hydrogels for neuroprotection, and (4) encapsulation of drugs to increase erectile function [72]. Topical delivery and encapsulation are feasible approaches that can be implemented to deliver herbal molecules for erectile dysfunction. Encapsulating into nanoparticles allows transdermal delivery of the bioactive agents, which improve the safety profile and minimize the first-pass metabolism [73]. *Panax* *ginseng* C.A. Mey (Araliaceae) is one of the most popular herbs for the treatment of erectile dysfunction (Table 1) [49,51,74]. Ginsenoside, a steroid glycoside from *Panax ginseng* C.A. Mey (Araliaceae), was reported to demonstrate a direct effect on triggering an erection, which is mediated through the release of endothelial nitric oxide (NO) [75]. Formulation of cream containing nanoparticles of ginsenosides is a promising approach to provide an on-demand erection effect for patients with erectile dysfunction (Figure 3). The local delivery of the bioactive compound minimizes the systemic absorption of the compound into the bloodstream.

Urological disorders, especially urinary tract infections (UTIs), are common and affect over 150 million people worldwide every year [76]. According to the World Health Organization (WHO), around 50% of women experience a UTI at some point in their lives, but UTIs are present in men as well [77]. UTIs can be uncomplicated or complicated. Uncomplicated UTIs typically affect healthy persons with no other known disorders and these are further classified into lower (e.g., cystitis) and upper UTIs (e.g., pyelonephritis) [78]. Complicated UTIs occur when the urinary tract or host defense is compromised by disorders, including urinary retention, renal failure, or immunosuppression [79].

Gram-negative, Gram-positive bacteria, and some fungi are the causative agents of UTIs. Among them, the most common agent is the uropathogenic *Escherichia coli* (UPEC), which can be responsible for both complicated and uncomplicated UTIs. Other microorganisms causing uncomplicated UTI are *Klebsiella pneumoniae*, *Staphylococcus saprophyticus*, *Enterococcus faecalis*, *Proteus mirabilis*, *Pseudomonas aeruginosa*, *Staphylococcus aureus*, and *Candida* spp. [80,81]. Besides UPEC, *Enterococcus* spp., *K*. *pneumoniae*, *Candida* spp., *S*. *aureus*, *P*. *mirabilis*, and *P*. *aeruginosa* are mostly responsible for complicated UTIs [82,83]. Since UTIs are commonly treated with antibiotics, this can cause increases in antibiotic resistance and alterations in gut and vaginal microflora [84,85]. On the other hand, any proper vaccines against UTI-causing microorganisms are currently absent, leaving antibiotics as the only therapeutic choice. Searches are ongoing for new antibiotics or other phytochemicals from plant sources [86], but thus far without any significant development in this field.

The current situation regarding UTIs and drugs is a classic example of the scientists and researchers maybe needing to adopt the policy of “less is more”. This can be done through nanotechnological approaches. These approaches can be undertaken in several fields, such as application of nanotherapeutics (application of nanodrugs may result in greater efficacy and so lesser use of drugs and thus decreasing drug resistance), nanodelivery (which can not only improve drug absorption but also enable drugs to reach only target organs), nanodiagnostics (can reduce the need for invasive procedure diagnostics), and nanocarriers (for targeted delivery, sustained release, and allowing a longer time in circulation for drugs).

Polymeric nanoparticles (NPs) are emerging as suitable agents as nanotherapeutics due to their ability to accumulate onto cell membranes and then destroy bacterial cells, therefore producing an antibiotic effect. Moreover, NPs can differ from each other according to the needs and can be loaded with different drugs [87]. The antibiotic-like effects of antimicrobial polymers would depend on their chemical structures, which may be quaternary nitrogen groups, halamines, or polylysine. Various amphiphilic polymers have been evaluated against ESKAPE pathogens, which have been reviewed by Kamaruzzaman et al. [87]. A number of organic NPs have been tested against uropathogens as reviewed by Sánchez et al. [88]. Two of the NPs containing herbal components include polyphenol 60 and curcumin nanoemulsion-based gel for intravaginal use against UPEC, and polyphenol 60 plus cranberry nanoemulsion-based gel for intravaginal applications against *E. coli* [89,90].

The use of plants for biological synthesis of NPs containing inorganic elements or compounds (green NPs) is another rapidly developing area for the use of NPs against UTI pathogens. The leaf extract of *Azadirachta* *indica* A.Juss (Meliaceae) has been used to synthesize Ag-embedded mesoporous silica nanoparticles (mSiO_2_-AgNPs) against *Candida* *albicans* [91]. AgNPs, synthesized with the plant *Anogeissus* *acuminata* Wall. (Combretaceae), when tested against 11 multidrug-resistant (MDR) pathogens isolated from UTI patients (*Staphylococcus* *aureus*, *Enterococcus* *faecalis*, *Acinetobacter* *baumannii*, *Citrobacter* *freundii*, *Enterobacter* *aerogenes*, *Escherichia* *coli*, *Klebsiella* *oxytoca*, *Klebsiella* *pneumoniae*, *Proteus* *mirabilis*, *Proteus* *vulgaris*, and *Pseudomonas* *aeruginosa*) demonstrated good effects against the pathogens [53]. Green copper-based NPs using leaf extract of *Camellia* *japonica* L. (Theaceae) inhibited the growth of the uropathogens *Klebsiella* *pneumoniae* and *Pseudomonas* *aeruginosa* [92]. Green NPs have the advantage of not only containing antimicrobial compounds or extracts, but the plant or plant part extract used to produce the NPs may have antimicrobial activity itself.

Catheters are a major cause of UTIs. It has been found that coating catheters with NPs of essential oils (Eos) such as tea tree oil or EO components such as terpinen, cineole, and eugenol can protect against *Proteus* *mirabilis* biofilm formation [93]. Taken together, alternative medicinal systems can be expanded to incorporate the nanotechnological application of plant-based nanomaterials for providing not only new but better treatments for UTIs, as well as producing preventive techniques against the development of UTIs in the first place. Besides the green silver and copper-based NPs mentioned above, there are other examples of new developments in this field of metal-plant-based nanoparticle therapeutics. Zinc oxide nanoparticles (ZnO-NPs) synthesized from the leaves of *Berberis* *aristata* DC. (Berberidaceae) have been found effective against *Escherichia coli*, *Staphylococcus aureus, Klebsiella pneumoniae, Bacillus subtilis, Bacillus cereus*, and *Serratia marcescens* [94]. Zinc oxide nanoparticles synthesized from leaves of *Passiflora caerulea* L. (Passifloraceae) demonstrated inhibitory activity against several uropathogens [54]. Copper nanoparticles (Cu-NPs) of *Cissus* *vitiginea* L. (Vitaceae) showed efficacy against several UTI pathogens such as *E. coli*, *Enterococcus* sp., and *Klelbsiella* sp. [95]. Silver (Ag)-NPs *Mimosa* *pudica* L. (Fabaceae) alcoholic extracts and *Nigella* *sativa* L. (Ranunculaceae) seeds have excellent antibacterial effects on UPEC and *Staphylococcus aureus* and other UTI pathogens [56,57,96]. Noticeably, Badiger and colleagues mentioned that cranberry extract is the only supplement effective against UTIs along with antibiotics [96].

Therefore, it is quite feasible that green NPs can be the UTI therapeutics of the future. Not only can they be easily administered, but despite their metallic content, they are less toxic than conventional medicines due to the extremely low concentrations involved. Moreover, they provide an alternative to antibiotic-resistant UTI pathogens. The plant coatings of the NPs can be antibacterial by themselves, so every green NP containing a metal antibacterial or other antibacterial component(s) would contain two different antibacterial components, and as such, raise their efficacy. Despite the immense promises of nanotechnology, it is still basically a research topic and less an applied subject in the field of UTI therapeutics. It is expected that this situation will change in favor of NP application to treat UTI infections sooner rather than later. Appropriate herbal extracts in green NPs will facilitate their use against multidrug-resistant pathogens [97].

### 3.3. Nano-Phytopharmaceuticals in Locomotor Disorders

Nano-phytochemicals are being used widely that are not only limited to dermatological or urological disorders, but also movement or coordination disorders. Movement disorders are neurological conditions with either an excess of movement (hyperkinesias) or a paucity of voluntary and automatic movements, unrelated to weakness or spasticity (hypokinesias) [98,99,100]. Long-term opioid intake can cause motor behavioral disorders like hyperkinesias and hypokinesias, and it is more prevalent in patients with chronic pain, and who need to take opioids to relieve pain [101,102,103]. Abnormalities in the upper motor neurons, lower motor neurons, neuroinflammation, and/or the effector muscle tissues are known to be underlying factors. In this section, we discuss a few examples of natural products that were reported to have an ameliorative effect on animal or clinical models of movement disorders and the potential applications of nanoformulations to enhance their biomedical application. It is noteworthy that the reports on nano-phytoformulations on locomotor disorders are scarce; thus, the potential applications should be further explored.

*Mucunapruriens* (L.) DC. (Fabaceae), commonly known as “Mucuna” or “velvet bean” is an annual legume of the family Fabaceae. It is known to have a variety of therapeutic effects, such as anti-oxidant, anti-inflammatory, anti-epileptic, antimicrobial, and aphrodisiac effects [104]. Among all the bioactive substances found in *Mucuna pruriens* (L.) DC. (Fabaceae) seed extract, L-DOPA, the precursor of dopamine, constitutes almost 5% of its total phytochemical content [105]. This distinctive feature of *Mucuna pruriens* (L.) DC. (Fabaceae) seed extract was used as the complementary therapy for Parkinson’s disease (PD), in animal models and clinical settings [106]. Clinical studies have demonstrated that a single dose [107], but not prolonged 16-week treatment with *Mucuna pruriens* (L.) DC. (Fabaceae) seed extracts caused significant motor improvement in PD patients, comparable with conventional levodopa medication [108]. The application of prolonged *Mucuna pruriens* (L.) DC. (Fabaceae) seed extract was hampered by the emergence of gastrointestinal side effects [108]. Nonetheless, in developing countries conventional levodopa medication is too costly, hence the *Mucuna pruriens* (L.) DC. (Fabaceae) seed extract could be regarded as a potential source of natural L-DOPA particularly by implementation of nanotechnology to reduce its potential side effects [109]. Further study on identifying the other possible bioactive compound(s) synergizing with natural L-DOPA in *Mucuna pruriens* (L.) DC. (Fabaceae) seed extract, without the aforementioned gastrointestinal side effects, would benefit the future pharmacological development of this natural product. Given that another clinical study demonstrated that oral administration of *Mucuna pruriens* (L.) DC. (Fabaceae) seed extract exerted rapid onset of action and longer efficacy in PD patients without the increase in dyskinesias, a common side effect of conventional levodopa medication, it is suspected that other bioactive substances were playing a pivotal role in the PD-relieving effect of *Mucuna pruriens* (L.) DC. (Fabaceae) seed extract [110]. Indeed, such observation was also replicated in nonhuman primates, suggesting another distinct mechanism of action besides the dopaminergic supplementation via L-DOPA [111]. Another study showed that L-DOPA deprived *Mucuna pruriens* (L.) DC. (Fabaceae) seed extract could also significantly exert a neuroprotective effect in an in vitro model of PD [112]. Taking into consideration that synergism among bioactive components in *Mucuna pruriens* (L.) DC. (Fabaceae) seed extract is crucial for its anti-PD effect, the methanolic extract (0.2 g’kg, intraperitoneal (i.p.)) of *Mucuna pruriens* (L.) DC. (Fabaceae) seeds produced 1-methyl-4phenyl-1, 2, 3, 6- tetrahydropyridine-induced neurotoxicity and motor behavioral toxicity in a mouse model of PD [113]. Noticeably, a single daily gold nanoparticle incorporated in the methanolic extract of *Mucuna pruriens* (L.) DC. (Fabaceae) supplementation (0.5-20.0 mg/kg/day i.p., for 7 days) prevented motor behavioral neurotoxicity more efficiently than the effects of the methanolic extract of *Mucuna pruriens* (L.) DC. (Fabaceae) [113]. In this study, motor behavioral toxicity was measured using rotarod, narrow beam walking and hang test [113].

Apart from *Mucuna pruriens* (L.) DC. (Fabaceae)*,* *Moringa* *oleifera* Lam. (Moringaceae) that is known as “mironga’” or “drumstick tree”, is a member of the Moringaceae family, widely cultivated in tropical and subtropical regions. Its leaves and fruits are commonly consumed as food as well as herbal medicine in Ayurvedic and traditional Chinese medicine, for their anti-diabetic, anti-cancer, anti-inflammatory, and anti-oxidant properties [111,114,115]. Furthermore, literature on the effect of *Moringa oleifera* Lam. (Moringaceae) leaves extract in alleviating central and peripheral movement disorder is available on preclinical models [116,117]. In rats induced with focal ischemic stroke, the stroke-associated-motor impaired condition, e.g., hypolocomotion and stereotypic behaviors, was significantly suppressed by oral administration of *Moringa oleifera* Lam. (Moringaceae) leaves extract for 7, 14, and 21 days. In a Parkinson’s disease mimicking model, a single oral administration of *Moringa oleifera* Lam. (Moringaceae) leaves extract was reported to reverse the haloperidol-induced catalepsy in mice, measured by akinesia and rigidity responses [118]. In another sub-chronic model of PD in mice induced by the neurotoxin 1-methyl-4-phenyl-1,2,3,6-tetrahydropyridine, 10-day repeated treatment with moringin, the bioactive substance isolated from *Moringa oleifera* Lam. (Moringaceae) leaves extract, significantly ameliorated the PD-like motor deficits and bradykinesia. In another study that used the sciatic nerve injury-induced muscle atrophy as a peripheral model of motor impairment, mice fed with *Moringa oleifera* Lam. (Moringaceae) leaves extract-enriched chow for 14 days showed a significant muscle mass and motor grip force restoration, as compared with the normal chow control group [119]. However, another report showed that similar doses of *Moringa oleifera* Lam. (Moringaceae) leaf extract, administered via the oral route, exerted CNS depressant and muscle relaxant effects [120], suggesting possible opposing effects among bioactive compounds of *Moringa oleifera* Lam. (Moringaceae) leaves extract. Nonetheless, these data warrant further research on the varied contribution of each isolated compound in *Moringa oleifera* Lam. (Moringaceae) leaves extract, possibly on both hyperkinesias and hypokinesias-related movement disorders. Besides its therapeutic effect in animal models, *Moringa oleifera* Lam. (Moringaceae) leaves extract has been reported to be used as a reducing agent in the green biosynthesis of silver nanoparticles, owing to its strong antioxidant effect [121,122]. Interestingly, the nanoformulation that incorporates *Moringa oleifera* Lam. (Moringaceae) leaves extract with silver nanoparticles showed enhanced biological activities such as antioxidant, cytotoxic, and free radical scavenging activity [123]. The effects of nano-formulations of *Moringa oleifera* Lam. (Moringaceae) leaf extract or its bioactive compound, moringin, can be investigated in animal models of movement disorder and the outcome of this study can provide its suitability in future clinical use. Kolaviron, a natural flavonoid obtained from the seeds of *Garcinia kola* Heckel (Guttiferae) that is found in Cameroon and some other African countries, is used as a traditional medicine to treat the common cold, coughs, fever, and similar diseases [124]. Kolaviron showed anti-inflammatory, anti-colitis, anti-oxidant effects and prevented genotoxicity [124,125]. Kolaviron prevented multi-walled carbon nanotubes (MWCNTs)-induced neurotoxicity (as shown with reduced locomotor activities) in rats [124]. In this study, 10-week-old male Wistar rats treated with kolaviron (100 mg/kg/day, oral) over a period of 15 days prevented neurotoxicity (defined as reduced exploratory and locomotor activities, such as total distance traveled, increased horizontal and turning behavior) induced by MWCNTs (1.0 mg/kg/day, intraperitoneal injections). Additionally, kolaviron treatment showed some neuroprotective effects as shown by their histological analysis of the brain [124].

## 4. Conclusions

Nano-encapsulation of herbal extracts and phytoconstituents has been reported as an outstanding strategy to overcome current challenges associated with herbal medicines, such as lower solubility, less target specificity, less bioavailability, and shelf life. A number of studies have reported the successful use of nano-phytopharmaceuticals as therapeutic agents for locomotor disorders, dermatological, and urological disorders in vivo and in vitro. Nanotechnology has been reported to improve the physicochemical properties, efficacy, and bioavailability of the herbal medicines used in locomotor, dermatological, and urological disorders. However, available data in this research field is mostly from preclinical, in vivo, and in vitro studies over short-term observations. Physicochemical characteristics of nano-phytomedicines that modify their in vivo efficacy should be validated in future studies to knock over current impediments in this research and development of rationally designed nano-phytomedicines for clinical studies. Moreover, a focus on clinical translation studies is warranted, such as pharmacokinetics and long-term toxicity, to address the important questions regarding the clinical feasibility of nano-phytomedicine in locomotor, dermatological, and urological disorders. In the future, it is expected to have meaningful development in nanoparticles-based phytomedicines as an essential aspect of human health management.

## Figures and Tables

**Table 1:** Role of nano-phytopharmaceutical formulations against various locomotor, skin, and urogenital disorders.

Plant Source	Formulation	Study Type	Action	Reference
Citrus fruits, onions, apples, parsley, sage, tea, and berries.	Nanoencapsulated quercetin in zein nanoparticles (NPQ)	Preclinical (rats)	NPQ improved memory and cognitive ability in rats (but no effects on locomotor activity test)	[37,38]
Citrus fruits, onions, apples, parsley, sage, tea, and berries.	Quercetin nanoparticles	Preclinical (rats)	Quercetin nanoparticles improved memory and pathological damage induced by scopolamine	[39,40]
Berries, currants, grapes, red to purplish blue colored leafy vegetables, grains, roots, and tubers.	Anthocyanin-loaded poly (ethylene glycol)-gold nanoparticles (PEG-AuNPs)	Preclinical (mice)	PEG-AuNPs improved amyloid-beta (Aβ_1__-__42_) induced neuronal damage and neuroinflammation	[41,42]
*Curcuma longa* L. (Zingiberaceae)	Nano-curcumin particles	Preclinical (mice)	Enhanced memory, motor function, contextual fear	[43]
*Anamirtacocculus* (L.) Wight and Arn. (Menispermaceae)	*A.cocculus* NPs in cocc 30c, in a homeopathic formulation	Preclinical	Improved attention and motor functions in sleep-deprived rats	[44]
*Solanum tuberosum* L. (Solanaceae)	*S.tuberosum* Lectin NPs	Preclinical	Helped improved drug delivery enhanced memory and motor function	[45]
*Azadirachta* *indica* A.Juss. (Meliaceae)	Neem oil incorporated in argan-liposomes and argan-hyalurosomes by sonicating with argan oil, soy lecithin, and water	In vitro	Protected skin cells by reducing oxidative stress	[46].
*Curcuma* *longa* L. (Zingiberaceae)	Curcumin formulated with lipid-based nanoparticles such as liposomes, niosomes, solid lipid nanoparticles, and nanostructured lipid carriers	Review	Improved its penetration into skin and thus increased the solubility, stability, and therapeutic efficiencies of curcumin against various dermatological disorders such as psoriasis, dermatitis, bacterial, viral and fungal infections, burns, acne, arthritis, and skin cancer	[33,34]
*Curcuma longa* L. (Zingiberaceae)	*C. longa* leaves extract Silver nanoparticles (CL-AgNPs) loaded cotton fabric	In vitro	Enhanced wound healing and antimicrobial activity on skin	[47]
*Curcuma longa* L.(Zingiberaceae)	Solid lipid nanoparticles (SLN-curcuminoids)	Ex vivo (Sheep ear skin)	Showed good spreadability and stability on skin	[48]
*Curcuma longa* L. (Zingiberaceae)	Curcumin nanoparticles (curc-NPs)	Preclinical (rats)	Improved erectile response in diabetic male rats	[49,50]
*Panax ginseng* C.A. Mey (Araliaceae)	*P.ginseng* nanoparticles	Preclinical (rats)	Improved serum testosterone secretion and decrease sperm abnormalities in male rats	[51]
*Oxaliscorniculata* L. (Oxalidaceae)	Aqueous extract of *O.* *corniculata* and its biofabricated silver nanoparticles (AgNPs)	In vitro	Effective against urinary tract infection (UTI) causing microorganisms	[52]
*Anogeissusacuminata* *Wall.(Combretaceae)*	Aqueous leaf extract of *A. acuminata* and its AgNPs	In vitro	Effective against multidrug resistant UTI causing bacteria	[53]
*Passiflora caerulea* L. (Passifloraceae)	Zinc oxide nanoparticles (ZnO NPs) using *P. caerulea* extract	In vitro	Effective against multidrug resistant UTI causing bacteria	[54]
*Catharanthus roseus* *(*L*.)* G. Don (Apocynaceae)	Sulphur nanoparticles (SNPs) produced from *C. roseus* leaf extract	In vitro	Effective against multidrug resistant UTI causing bacteria	[55]
*Mimosa* *pudica* L. (Fabaceae)	Sulphur nanoparticles (SNPs) produced from *M. pudica* alcoholic extracts	In vitro	Antibacterial effects on uropathogenic *E*. *coli* (UPEC) and *S. aureus* and other UTI pathogens	[56]
*Nigella* *sativa* L. (Ranunculaceae)	Sulphur nanoparticles (SNPs) produced from seeds of *N. sativa* L. alcoholic extracts	In vitro	Antibacterial effects on UPEC and *S. aureus* and other UTI pathogens	[57]
*Rauwolfia* *serpentina* L. (Apocynaceae)	Biologically synthe-sized gold nanopar-ticles with aqueous leaf extract of *R. serpentina* L.	In vitro	Antibacterial effects on *E. coli* and *S. aureus*	[58]
